# Hypoglycaemia in older home-dwelling people with diabetes- a scoping review

**DOI:** 10.1186/s12877-020-01961-6

**Published:** 2021-01-07

**Authors:** Monica Hermann, Lovise S. Heimro, Anne Haugstvedt, Ingvild Hernar, Arun K. Sigurdardottir, Marit Graue

**Affiliations:** 1 Faculty of Health and Social Sciences, Institute of Health and Caring Sciences, Western Norway University of Applied Sciences, Stord, Norway; 2 Faculty of Health and Social Sciences, Institute of Health and Caring Sciences, Western Norway University of Applied Sciences, Bergen, Norway; 3 School of Health Sciences, University of Akureyri, Akureyri, Iceland; 4 Akureyri Hospital, Akureyri, Iceland

**Keywords:** Diabetes, Older adults, Hypoglycaemia, Home-dwellers, Scoping review

## Abstract

**Background:**

Hypoglycaemia is associated with cognitive and functional decline in older people with diabetes. Identification of individuals at risk and prevention of hypoglycaemia is therefore an important task in the management of diabetes in older home-dwelling individuals. The purpose of this scoping review was to map the literature on hypoglycaemia in home-dwelling older people with diabetes.

**Methods:**

This scoping review included original research articles on hypoglycaemia in older (≥ 65 years) individuals with diabetes from developed countries. A broad search of the databases Cinahl, Embase and Medline was performed in July 2018. The report of the scoping review was conducted in accordance with the PRISMA Extension for Scoping Reviews.

**Results:**

Our database search identified 577 articles of which 23 were eligible for inclusion. The identified literature was within four areas: 1) incidence of hypoglycaemia in older home-dwelling people with diabetes (11/23 articles), 2) risk factors of hypoglycaemia (9/23), 3) diabetes knowledge and self-management (6/23) and 4) consequences of hypoglycaemia for health care use (6/23). The majority of the literature focused on severe hypoglycaemia and the emergency situation. The literature on diabetes knowledge and management related to preventing adverse events relevant to older home-dwellers, was limited. We found no literature on long-term consequences of hypoglycaemia for the use of home health care services and the older persons’ ability to remain home-dwelling.

**Conclusions:**

We identified a lack of studies on prevention and management of hypoglycaemia in the older individuals’ homes. Such knowledge is of utmost importance in the current situation where most western countries’ governmental policies aim to treat and manage complex health conditions in the patient’s home. Future studies addressing hypoglycaemia in older individuals with diabetes are needed in order to tailor interventions aiming to enable them to remain home-dwelling as long as possible.

**Supplementary Information:**

The online version contains supplementary material available at 10.1186/s12877-020-01961-6.

## Background

Diabetes is one of the major public health challenges of our time. Globally, diabetes is expected to increase by around 50% to 700 million people within 25 years, and the prevalence rises with increasing age [1, 2]. As the risk of diabetes and associated complications increases with age, rates of hypoglycaemia, cardiovascular complications, and mortality increase steeply with advancing age [3]. Thus, in an aging population the number of older home-dwelling people with diabetes in need of health services is increasing. A study among older people receiving home care in a Norwegian municipality indicated a diabetes prevalence of 26%, whereof 14% of the individuals were not aware of their diagnosis [4].

Hypoglycaemia is a well-known side-effect of diabetes treatment. Episodes of severe hypoglycaemia, defined as episodes requiring assistance of others, are associated acute adverse events, such as seizures, coma, falls/fractures, and long term consequences, such as physical and cognitive dysfunction [5]. Episodes of severe hypoglycaemia also increase the risk of major cardiovascular events, cardiovascular death and all-cause mortality [5, 6]. The risk of hypoglycaemia increases with increasing age and the consequences for older people with diabetes may even be more severe, because falls and fractures are often followed by functional decline [7]. Age-related physiologic changes causing an increased risk of hypoglycaemia include changes in counter-regulatory hormones, lack of autonomic warning symptoms and pharmacokinetic changes leading to increased drug response [8–11]. Impaired cognitive function, poor nutritional, status, polypharmacy and frailty are additional factors contributing to increased risk of hypoglycaemia in older people [12, 13]. Also, the similarity between symptoms of hypoglycaemia and symptoms of dementia, such as confusion, agitation and behavioural changes, may lead to missed diagnosis of hypoglycaemic episodes in older people [14]. Reduced cognitive function has been linked to increased risk of hypoglycaemia and vice versa [15]. The general recommendation on glycaemic control in older individuals is therefore that hypoglycaemia should be avoided in order to prevent further cognitive and functional decline and other major adverse outcomes, such as cardiovascular events [16].

In order to achieve and maintain glycaemic control, and prevent both hyper- and hypoglycaemia, current guidelines recommend frequent glucose monitoring in individuals using insulin and insulin secretagogues drugs [7, 17, 18]. However, the value of self-monitoring blood glucose in non-insulin users has been a matter of debate [19, 20]. Older individuals often rely on family members or other caregivers to follow-up blood glucose monitoring and to recognize and manage episodes of hypoglycaemia. Inadequate knowledge of the caregiver could put the older individual at increased risk of adverse events [21].

The increasing number of older people with diabetes and their complex need for follow-up in order to prevent adverse events, imply an increasing number of older people with diabetes in need of health services. In Norway, more than 10% of individuals above 80 years of age receive home care services and for individuals above 90 years, the fraction exceeds 25% [22]. For older people with diabetes, the home care services often include help with administration of medication, blood glucose monitoring and follow-up of diabetes-associated complications. We have previously demonstrated a significant discrepancy between diabetes guideline recommendations and clinical practice with respect to glucose monitoring, individualized treatment goals for Haemoglobin A1_c_ (HbA1c) and documentation in nursing homes [23]. Similar findings are reported for individuals receiving home care [24]. Inadequate follow-up of guidelines may put older people at risk of adverse events, such as hypoglycaemia.

Although there is a fair amount of research on diabetes management in nursing homes [25], far less is known about older home-dwelling individuals and their complex needs for help with diabetes management in order to prevent hypoglycaemia. Therefore, there is a need for a knowledge base on current practices related to hypoglycaemia in older home-dwelling individuals with diabetes, to serve as a guide in the development of future research aiming to increase the quality of care for older people with diabetes. Good quality care is essential to conserve health and functioning and further increase the older person with diabetes’ ability to remain home-dwelling. On this basis, the present scoping review was performed to identify and map the literature on hypoglycaemia in home-dwelling older people with diabetes.

## Methods

Three members of the research team (MH, AKS and MG) drafted the protocol for this scoping review based on the scoping review methodology described by Arksey and O’Malley and the further refinement by Levac et al. [26, 27]. The protocol can be obtained by contacting the corresponding author. The report of the scoping review was conducted in accordance with the PRISMA Extension for Scoping Reviews [28].

### Information sources and literature search

We searched the databases Cinahl, Embase and Medline to identify relevant studies. The search strategy was developed in collaboration with an experienced research librarian, who also performed the search. A broad search on hypoglycaemia in older home-dwelling people with diabetes was performed July 2nd, 2018. To ensure identification of all relevant studies, we included search terms for community-care, long-term care as well as home care as the spectrum of community-based health care is broad and varies between different countries. In the further selection process, only papers reporting data on older people living independently in their own home (i.e. home-dwelling) were included. Papers on older people living in any kind of care facilities or senior citizen complexes with available assistance (i.e. community-dwelling) were not included. Further, we included search terms on hypoglycaemia as well as antidiabetic agents known to cause hypoglycaemia. The search included the following keywords/MeSH-terms: ‘Diabetes Mellitus’, ‘diabetes type 1’, ‘diabetes type 2’, ‘hypoglycaemia’, ‘antidiabetic agent’, ‘home health care’, ‘community health service’, ‘home care’, ‘nursing home’, ‘long term care’. We conducted a comprehensive search with no language or date limit. The final electronic search strategy for Medline can be found in supplementary file 1. To supplement the search, we searched the reference lists of included articles for relevant studies and searched for further publications by the researchers identified in the included articles.

### Eligibility criteria and study selection

All identified abstracts from the databases were transferred to an excel file and duplicates were excluded. Two members of the research team (MH and MG) independently screened titles and abstracts against a set of minimum inclusion criteria. If the reviewers disagreed, or if the abstract did not contain enough information to determine eligibility, the article was examined in full text before the reviewers reached consensus. The criteria were tested on 5% of the abstracts to ensure that they were relevant to capture the scope of this review. Papers on hypoglycaemia in older (age ≥ 65) home-dwelling people with diabetes originating from developed countries (Japan, Canada, US, Australia, New Zealand and Europe, excluding former Yugoslavia, the Russian Federation, Ukraine and Belarus) and written in English, Danish, Swedish or Norwegian language were eligible for inclusion. We allowed inclusion of studies with any type of study design. Studies reporting only on health care professionals caring for people with diabetes and studies reporting only on younger individuals (age < 65), were excluded. Also, review articles, commentaries, editorials, or other publications not based on empirical data, were excluded. Abstracts eligible for inclusion were further reviewed in full text by two members of the research team (MH and MG) and articles that did not meet the above-mentioned inclusion criteria were excluded.

### Data charting process

A data extraction template was developed to ensure that all data relevant for the objective of the scoping review was included. The data extraction chart comprised publication year, publication type, study design, setting, type of data, year of data collection, age and number of patients, study objective and results relevant for the objective of the scoping review. The data chart was tested and further refined by two researchers (MG and MH). The full data charting process was done by five of the authors in pairs (IH, AH, LSH, MG and MH). The researchers in each pair first independently extracted data from the full-text articles, before the two researchers in each team compared their data extraction. Discrepancies were discussed in the research team to ensure consistency between the extracted data from all studies.

### Results

**Fig. 1:**
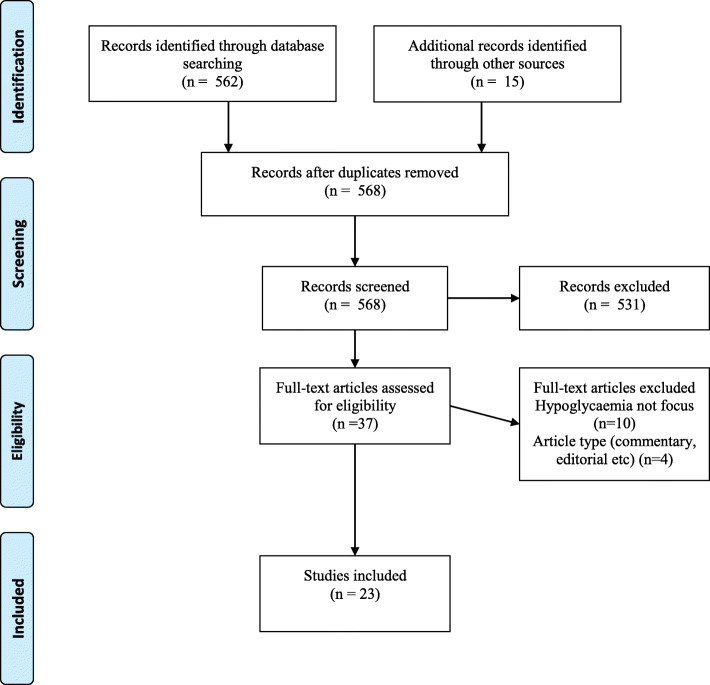
Selection of sources of evidence

A total of 577 articles were retained from our initial search (Fig. 1). After removing nine duplicates the abstracts of the remaining 568 articles were screened applying the exclusion criteria. Full-article screening was performed for 37 articles, of which 23 were included in the study. The studies’ place of origin, study population, setting, design, and focal areas are presented in Table 1. Data from the individual sources of evidence were within the following areas: 1) incidence of hypoglycaemia in older home-dwelling people with diabetes, 2) risk factors of hypoglycaemia, 3) diabetes knowledge and self-management, and 4) consequences of hypoglycaemia for health care use (Table 1).

**Table 1:** Summary of characteristics of the individual sources of evidence

First author, year (reference)	Place of origin	Population (n)	Age range	Setting	Study design	Data collection	Thematic focus/aim
**Dunning, 2005** [29]	Australia	T2DM^a^ (30)	33–84	Diabetes outpatient education centre	Cross-sectional	Structured interviews/observation of skills	Patient knowledge and self-management
**Farmer, 2012** [30]	UK	DM^b^ (4081)	45–77^d^	Ambulance calls	Retrospective observational	Records of emergency call assistance	Incidence, consequences
**Feil, 2011** [31]	US	DM and dementia/cognitive impairment (497,900)	> 65	Research database (home-dwellers and nursing home)	Cross-sectional	Research database	Incidence, risk factors
**Gehlaut, 2015** [32]	US	T2DM (108)		Endocrinology centre	Prospective observational, non-blinded	Continuous glucose monitoring	Incidence
**Hambling, 2017** [33]	UK	T2DM and chronic kidney disease (1379)	73–83^d^	General practice	Cross-sectional	Medical records/audit	Risk factors
**Harsch, 2018** [34]	Germany	T2DM (160)	47–101	Hospitalized patients	Retrospective survey	Standardized questionnaire and tests	Patient knowledge
**Hewitt, 2010** [35]	UK	T2DM (1047)	75–100	General practice	Factorial cluster randomized trial	Interview and standardized tests	Patient knowledge and management
**Holstein, 2003** [36]	Germany	DM (264)	4–95	Emergency admission to hospital	Cohort study	Clinical data (blood glucose measurements)	Incidence
**Holstein, 2010** [37]	Germany	T2DM (139)	46–97	Emergency admission to hospital	Prospective observational study	Clinical data (blood glucose measurements)	Incidence, risk factors
**Kachroo, 2015** [38]	US	T2DM (21613)	65–100	Commercial database	Retrospective cohort	Medical records	Consequences
**Lee, 2017** [39]	US	T2DM (1206)	64 (mean at baseline)	Ambulance calls, emergency department visits, hospitalization	Prospective cohort study	Medical records	Incidence, risk factors
**Lipska, 2013** [40]	US	T2DM (9094)	60 ± 10^e,f^	Community setting	Cohort study	Registry data and questionnaire	Risk factors
**Lipska, 2015** [41]	US	DM (1288)	73 ± 6^e^	Community setting	Cross-sectional	Registry data	Risk factors
**Parsaik, 2013** [42]	US	T2DM	63 ± 12^e^	Ambulance calls	Cohort study	Ambulance records	Incidence, consequences
**Penfornis, 2015** [43]	France	T2DM and renal disease (980)		Outpatients (GP and diabetologist)	Cross-sectional	Clinical data registered by general practitioner	Risk factors
**Rajendran, 2015** [44]	UK	T1DM^c^ (59) and T2DM (106)	47–84	Emergency department care	Cross-sectional	Electronic patient records	Incidence, consequences
**Reed, 2003** [45]	UK	DM (130)		Outpatients	Cross-sectional	Observations and structured questionnaire	Knowledge, self-management
**Reifegerste, 2016** [46]	Germany	Informal caregivers of T2DM patients (488)	61 ± 14^e^	Outpatients (participants of disease management program)	Cross-sectional	Survey	Informal caregiver knowledge
**Sotiropoulos, 2005** [47]	Greece	T2DM (207)	45–88	Hospital	Cross-sectional	Patient records, blood samples, questionnaire	Incidence, knowledge, risk factors
**Thomson, 1991** [48]	UK	T2DM (45)	61–82	Hospital	Case-control	Structured interviews	Knowledge
**Thorpe, 2015** [49]	USA	T2DM and dementia (15,880)	≥65	Mixed setting of home-dwellers, nursing home, hospital	Longitudinal retrospective cohort study	Patient records, registry data	Risk factors
**Villani, 2016** [50]	USA	DM (12,411)	57 ± 21^e^	Ambulance service	Retrospective observational study	Ambulance reports	Incidence, consequences
**Zaccardi, 2016** [51]	Australia	DM (79,172)	72% were > 60	Hospital admission	Longitudinal retrospective observational study	Hospital database	Incidence, consequences

^a^T2DM, type 2 diabetes mellitus

^b^DM, diabetes mellitus

^c^T1DM, type 1 diabetes mellitus

^d^interquartile range

^e^mean ± standard deviation

^f^age of informal caregivers

### Incidence of hypoglycaemia in home-dwelling older people with diabetes

In total, 11 studies reported on the incidence of hypoglycaemia in home-dwelling older people (Table 1) [30–32, 36, 37, 39, 42, 44, 47, 50, 51]. The majority of the studies reported data from ambulance records or emergency hospital visits [30, 36, 37, 39, 42, 44, 47, 50, 51], while one reported combined data of episodes of hypoglycaemia from outpatient visits, emergency departments and inpatient encounters with admission diagnosis hypoglycaemia [31]. Only one study reported observational data of mild, moderate and severe hypoglycaemia in home-dwelling older individuals registered by continuous glucose monitoring (CGM) [32].

Most studies reported incidences of hypoglycaemia requiring prehospital emergency medical care with or without hospital admission in patients with diabetes mellitus from 0.5–2.5 episodes/100 patient years [30, 36, 39, 44, 50, 51]. The incidence of hypoglycaemia reported by Parsaik and colleagues with data from the US was somewhat higher; ambulance calls related to hypoglycaemia were performed in 9/100 individuals with type 2 diabetes and the calls for hypoglycaemia were 2% of the total number of calls for emergency assistance [42]. Most studies reported combined data on type 1 and type 2 diabetes and did not separate on insulin users and patients using insulin secretagogues drugs or other glucose-lowering medication. However, Holstein et al. showed that the annual rate of severe hypoglycaemia requiring emergency medical service was 1.5 episodes per 100 individuals for insulin-treated individuals with type 2 diabetes, while the corresponding number for the overall group of patients with type 2 diabetes was 0.4 episodes per 100 individuals [36]. In a study reporting continuous glucose monitoring data in 108 individuals for 5 consecutive days, 49% had at least one hypoglycaemic event (defined as glucose level below 3.9 mmol/L or 70.2 mg/dL), while 21% had at least one severe hypoglycaemic event (defined as glucose level below 2.8 mmol/L or 50.4 mg/dL) [32]. In 75% of the individuals, at least one asymptomatic hypoglycaemic episode was observed. One or more non-insulin secretagogues drugs was the only glucose lowering medication in 18% of the individuals, while the remaining used insulin alone or combination therapy including either insulin or an insulin secretagogues drug. The sample included 38.7% individuals 65 years or older, but age specific analysis was not performed [32].

### Risk factors of hypoglycaemia in older home-dwelling people with diabetes

In total, 9 articles reported data on risk factors of hypoglycaemia in older home-dwelling individuals (Table 1) [31, 33, 37, 39–41, 43, 47, 49]. Factors contributing to the risk of hypoglycaemia included treatment-related factors (e.g. type of treatment, treatment intensity, and co-medication) and disease-related factors (e.g. comorbidity, functional status, and hypoglycaemia unawareness).

Several studies have shown that older individuals often are subjected to a tighter glycaemic control than recommended in guidelines, i.e. HbA1c below 53 mmol/mol (< 7.0%) [33, 41, 43, 49]. In the study by Lipska et al. as many as 62% had HbA1c below 53 mmol/mol and 43% had HbA1c below 48 mmol/mol (< 6.5%) [41]. However, the proportion of individuals with Hb1Ac below 53 mmol/mol (< 7.0%) did not differ across health status categories (relatively healthy, complex/intermediate health or very complex/poor health) [41]. The annual incidence of self-reported hypoglycaemia requiring assistance from others was 11% in individuals with type 2 diabetes, with no significant difference across different levels of glycaemic control [40]. However, there was a tendency towards higher incidence of hypoglycaemia in the groups with the lowest (below 42 mmol/mol or 6.0%) and highest (above 75 mmol/mol or 9.0%) HbA1c values. Also, in a study of almost 16,000 older individuals with type 2 diabetes and dementia, where the majority were home-dwellers, more than one-half had HbA1c levels of 53 mmol/mol or lower [49]. Advanced age (≥75) and weight loss were among the key risk factors for tight glycaemic control [49]. The most common glucose lowering drugs (either sulphonylureas alone or a combination of insulin and sulphonylureas) used in individuals with the lowest HbA1c, differed between studies [33, 41].

Several factors related to comorbidity also contributed to increased risk of hypoglycaemia in older home-dwellers. Among others, the risk of hypoglycaemic events was 1.6-fold and 2.4-fold higher in individuals with cognitive impairment and dementia, respectively [31, 39]. Also, the risk of hypoglycaemia was about 2-fold higher in older individuals with renal impairment [39]. There is some evidence of overtreatment in older individuals with renal impairment as indicated in a study of 980 individuals with type 2 diabetes and renal disease where the average HbA1c was 50 mmol/mol (6.7%) [43]. Also, among 139 cases of sulphonylurea-induced hypoglycaemia where 80% of the individuals were 70 years or older, 73% had renal impairment (estimated creatinine clearance below 60 ml/min) [37]. The extensive study from Lee et al. with 15 years follow-up data of 1206 individuals, showed a 2-fold risk of a severe hypoglycaemic event during the follow-up period among individuals with difficulties with activities of daily living or individuals using antidepressant medication [39]. Two studies have shown that a subgroup of patients with hypoglycaemia unawareness account for the majority of the repeated hypoglycaemic events [36, 40]. In the study by Lipska et al., where around 10% of the 9094 respondents had experienced severe hypoglycaemia, approximately 30% had four or more annual incidences of hypoglycaemia requiring assistance from others [40].

### Diabetes knowledge and self-management

Six of the included studies have investigated diabetes knowledge and/or self-management skills among older home-dwelling individuals [29, 34, 35, 45, 47, 48] (Table 1). While one study reported on information seeking and knowledge among informal caregivers of adults with type 2 diabetes [46].

In a study of 207 hospital admissions due to hypoglycaemia, around 85% had poor diabetes knowledge measured by a self-report questionnaire [47]. Chronic kidney failure was present in 22% of the individuals, and the most commonly reported cause of hypoglycaemia was missed meal (31%) [47]. Another study among people with diabetes ≥75 years in general practice, showed that 70–90% of insulin users were aware of the risk of hypoglycaemia, they knew how to act upon hypoglycaemia and 70% tested their blood glucose at home [35].

A study of knowledge and self-management skills in 30 individuals with type 2 diabetes of which 16 were insulin users, showed that a majority (28/30) reported having received some sort of education about their medication and diabetes management [29]. Education about when and how to take their medications were most commonly received (83%), while only 37% reported having received information about side-effects. A significant proportion of the participants demonstrated inadequate knowledge and self-management skills; 20% regularly forgot to take their medicines and 16% of insulin users did not dial up correct insulin dose. Among the 25 individuals who monitored their blood glucose at home, 20% did not performed control tests on their blood glucose meters [29]. A wide range of ethnic backgrounds were represented in the study and 40% had difficulties communicating without an interpreter [29]. In addition, Reed et al. reported corresponding data on self-management skills, where only 6% of insulin-treated individuals had no problems with blood glucose testing, meter use or insulin administration [45].

Individuals using oral medication with insulin secretagogues drugs seem to be less informed about their medication and side-effects compared to individuals on insulin. In a study of patients admitted to the emergency department with sulphonylurea-induced hypoglycaemia, 30% had received an education program [37]. Correspondingly, around 80% of the individuals treated with an insulin secretagogues drug (sulphonylureas or repaglinide) were not aware of the medications’ ability to cause hypoglycaemia and 57% did not know what to do if they experienced hypoglycaemia [34]. The proportion of individuals who knew how to respond on hypoglycaemia was similar (around 40%) for individuals using insulin secretagogues alone or in combination with insulin. Difference in knowledge between insulin users and users of peroral medication was also demonstrated by Thorpe et al., where awareness of the possibility of hypoglycaemia were reported by 68 and 12%, respectively [49].. However, the study did not differentiate between users of insulin secretagogues drugs and users of other oral antidiabetic medication.

We identified one study focusing on the knowledge of informal caregivers [46]. Among 488 informal caregivers to patients with type 2 diabetes, 1/4 of the caregivers could not list any symptoms of hypoglycaemia and 1/3 were not able to list any correct treatment for mild events of hypoglycaemia. Health professionals and print medias were reported as the main sources of information for informal caregivers [46].

### Consequences of hypoglycaemia for health care use in older home-dwelling people with diabetes

In total, 6 studies reported on the consequences of hypoglycaemia for use of health care services [30, 38, 42, 44, 50, 51]. All studies focused on emergency situations, such as transfer to an emergency department, admission to hospital, or length of hospitalization. We did not identify any studies on the use of home care or nursing home services after a hypoglycaemic event.

Several studies showed that hypoglycaemia in older home-dwelling individuals more often required hospitalizations than in younger individuals [30, 42, 50]. In the study by Farmer et al., 35% of the calls resulted in hospital admission, and the likelihood of being taken to hospital was almost double in individuals of 80 years or older compared to 20–60-year olds [30]. Villani et al. showed a 1.7-fold higher risk of transport to hospital for individuals above 75 years [50]. In addition, 60–70% of older insulin and/or sulphonylurea users were transferred to the emergency department and approximately 40% were further hospitalized with a 2 day median duration of stay [42]. Once transported to the emergency department, older individuals on sulphonylurea treatment more often had persistent hypoglycaemia (glucose below 3 mmol/L or 54 mg/dL) compared with insulin users (46% vs 22%) [44]. Older age has also been associated with longer hospital stays, along with female gender, and frailty/comorbidity [51]. Almost 1/5 of all patients admitted for hypoglycaemia had at least one previous admission for hypoglycaemia and 23% of the readmissions occurred within 1 month [51].

In the study by Rajendran et al., 10% of the hypoglycaemic events were accompanied by an additional serious harm, such as fall-related injury [44]. Elevated risk of fall-related events (fractures, head injuries and fall-related hospital admissions) has also been shown after discharge, with a 6-fold higher risk the first 30 days and a sustainable double risk for 1 year after a hypoglycaemic event [38]. Rajendran et al. also studied the long-term outcome following severe hypoglycaemia requiring hospitalization and found that 1 in 4 individuals died within the following year, with a mean time to death after discharge of 75 days [44].

## Discussion

In this scoping review we identified literature on hypoglycaemia in older home-dwelling people with diabetes within four areas; incidence of hypoglycaemia in older home-dwelling people with diabetes, risk factors of hypoglycaemia, diabetes knowledge and self-management, and consequences of hypoglycaemia for health care use. Most of the literature focused on incidence and risk factors of hypoglycaemia, while there was less literature on diabetes knowledge, self-management skills and consequences of hypoglycaemia in older home-dwelling individuals. Such knowledge is important in the current situation with strong governmental policies in most western countries to treat and manage complex health conditions in the patient’s home [52–54]. Knowledge on consequences of hypoglycaemia for the ability to remain home-dwelling, as well as diabetes knowledge and self-management skills, is important for planning future home health care services for this group of individuals.

The studies identified in this review reported incidences of hypoglycaemia requiring emergency medical care in older home-dwellers of 0.5–2.5 incidences/100 patient years [30, 36, 39, 44, 50, 51]. Farmer et al. showed that the incidence of hypoglycaemia was lower in older compared with younger adults, with an annual rate of 1.9% in individuals ≥65 years compared with an annual rate of 7.5% in individuals 15–35 years. Among older individuals, the risk of hypoglycaemia rises with increasing age as demonstrated in the study by Lipska et al. were rates of hypoglycaemia were 2-fold higher in individuals ≥75 years compared with individuals 65–74 years [55]. Although the annual rate among older people is lower than for younger individuals, the absolute number of severe events among older individuals is high due to the large proportion of older people with diabetes. Also, hypoglycaemia in older individuals more often require hospitalizations [30]. In light of the rising number of older people with diabetes, hypoglycaemia may be expected to contribute to an escalating burden on the health care services in the future. In all of the identified studies, hypoglycaemic events were registered through contact with the health care system, mostly emergency calls or admittance to hospital [30, 36, 39, 44, 50, 51]. We did not identify any studies reporting on hypoglycaemia managed by the person with diabetes, their next-of-kin or home care personnel. Although CGM data in older home-dwelling people with diabetes is scarce, there are indications of a much higher frequency of severe hypoglycaemia and undiscovered hypoglycaemia in older people than we have been aware of through epidemiological studies [56]. Gehlaut et al. found that hypoglycaemia was highly frequent, with 75% experiencing at least one asymptomatic hypoglycaemic event and 21% of the individuals experiencing severe hypoglycaemia during the 5 days of CGM data collection [32]. The risk of hypoglycaemia being unrecognised in older individuals has also been demonstrated in primary care where consultations due to non-specific symptoms were common in individuals over 65 years [57]. Although the literature so far mostly has focused on severe hypoglycaemic events requiring professional emergency health care, there are indications of relatively frequent hypoglycaemic episodes, including those of severity. This suggests that relying solely on epidemiological data and prescription records/ambulance calls may have led to an underestimation of the incidence of hypoglycaemia in older individuals.

The general recommendation on glycaemic control in older individuals is that hypoglycaemia should be avoided in order to prevent adverse outcomes [16]. The recent guideline from the American Diabetes Association recommends a glycaemic goal of < 58 mmol/mol (< 7.5%) in otherwise healthy older adults with few coexisting chronic illnesses and intact cognitive function and functional status [16]. Less stringent glycaemic goals (< 64*–*69 mmol/mol or < 8.0–8.5%) are recommended in individuals with multiple coexisting chronic illnesses, cognitive impairment, or functional dependence. Several of the studies identified in the present scoping review showed that older individuals are subjected to a tighter glycaemic control than recommended [33, 40, 41, 49]. Lipska et al. found that more than half of the older individuals with diabetes had HbA1c levels below recommended levels, and this also included individuals with very complex/poor health [41]. Similarly, in a large cohort of individuals with type 2 diabetes and dementia, more than half of the individuals were subjected to tight glycaemic control [49]. Also, Hambling and colleagues reported that 30% of a diabetes cohort had HbA1c below recommended levels, and there was no difference in median HbA1c between those with or without either chronic kidney disease or dementia [33]. These results indicate that the recommendations of less stringent glycaemic goals in order to reduce the risk of adverse events in older individuals with comorbidities, are often not followed.

Several studies suggest that older adults with elevated HbA1c are still at risk of hypoglycaemia [40, 58]. The indication that variance in blood glucose, rather than simply overall lower blood glucose, predispose older individuals to hypoglycaemia, is supported by a recent study from primary care [59]. Results from general practice records of almost 6000 individuals with type 2 diabetes showed that the prevalence of hypoglycaemia increased with increasing HbA1c [59]. Therefore, it is essential that clinicians recognize that elevated HbA1c does not protect against hypoglycaemia and that other efforts should be made to reduce the risk. Such efforts could include personalized treatment strategies to avoid overtreatment, use of medication with lower risk of hypoglycaemia, simplified treatment regimens and increased follow-up.

Individual risk factors, such as reduced renal function, cognitive impairment, difficulties with activities of daily living, use of antidepressants, and their association with risk of hypoglycaemia, were reported in several of the included studies [31, 33, 37, 39–41, 43, 47, 49]. In sum, many of the investigated individual risk factors increase the risk of hypoglycaemia by about 2-fold [31, 39]. Although there are some studies showing that the risk of hypoglycaemia is around 2-fold higher in older individuals with cognitive impairment/dementia or renal disease, there are no studies demonstrating causality. Also, we have not identified any studies where the combined and possible additive effect of the different risk factors have been assessed. This is highly important, as many older people suffer from several diseases and may have multiple risk factors affecting their risk for hypoglycaemia. Also, we did not identify any studies investigating association between risk of hypoglycaemia and co-habitation status. The potential effect of depression on diabetes management and hypoglycaemia was only reported in one study, which found a 2-fold increased risk of hypoglycaemia in individuals using antidepressant medication [39]. In sum, most of the literature focused on the effect of impaired cognitive function and/or renal function, while information on other risk factors was limited or lacking. For older home-dwelling persons with diabetes, the advice is to assess and reassess the patients’ risk profiles, as the appropriate balance between glycaemic control and risk of hypoglycaemia will change in line with changes in cognitive function, and thereby the individuals’ ability to follow up complex self-management tasks. Sufficient knowledge on risk factors is crucial in order to enable proper risk assessment, determine individual treatment goals and personalize treatment plans.

It is well known that severe hypoglycaemia may lead to seizures, coma, falls/fractures, as well as increased risk of major cardiovascular events and cardiovascular death [5, 6]. Less is known about the consequences of less severe episodes of hypoglycaemia (i.e., not leading to immediate hospitalization). However, common symptoms of transient falls in blood glucose in older people include confusion, dizziness and weakness, and there is a growing body of evidence that recurrent episodes of moderate hypoglycaemia increases the risk of functional decline and frailty in older people with diabetes [60]. Also, episodes of hypoglycaemia are potentially underreported due to non-specific symptoms and, for some individual, problems with communication of symptoms to health care providers. The consequence of undiscovered and/or misdiagnosed hypoglycaemia in older people with diabetes may be inadequate management of hypoglycaemic episodes. Thus, the risk of hypoglycaemia-related adverse events, such as falls and fractures may increase, possibly affecting the individuals’ long-term ability to self-manage and remain home-dwelling. However, we did not identify any studies exploring severe hypoglycaemia in older home-dwelling individuals and the long-term effects on use of health care services, need of home care, or the individuals’ ability to self-manage. With the rising number of older people with diabetes this is important to address. The increasing health care demands due to aging populations has led to many countries prioritising resources to facilitate that older individuals remain home-dwellers for as long as possible [52–54]. Concerning diabetes, hypoglycaemia is a potential threat to older individuals’ ability to continue residing at home. Therefore, early comprehensive monitoring of individuals risk factors and good quality home-care services, could be of importance for maintaining glycaemic control in older years and conserving the individuals’ ability for self-management, thereby enabling them to remain home-dwelling longer.

The lack of quality assessment of the included articles is a limitation of this scoping review. However, this is a general feature of the scoping review methodology, as the goal of a scoping review is simply to identify research that has been conducted, not necessarily to assess quality [26]. It must also be noted that there may be literature of relevance not included in this study, as only articles written in English, Danish, Norwegian, or Swedish were eligible for inclusion. It is a strength that no limitation on publication date was applied in the search strategy to ensure that relevant literature was not excluded. However, due to the instant and continuous development of diabetes treatment and follow-up, findings in some of the earlier articles may not be appropriate for the current situation. Also, services provided for home-dwelling older people with diabetes in their home vary in different countries and may also have changed during the last decades. Thus, the earliest work included in the review needs to be carefully interpreted. Also, we limited of origin of the studies in our search in order to identify studies from countries with somewhat comparable quality of health care system and follow-up of individuals with diabetes.

## Conclusion

The present scoping review revealed a fair amount of literature on the incidence of severe hypoglycaemia resulting in hospital admission in older home-dwelling adults, while there was very little data on episodes managed in the individuals’ homes. Also, different risk factors of hypoglycaemia have been studied independently, but given the complex situation of older home-dwelling individuals, there is a need for studies on the combined effect of risk factors in order to be able to identify individuals at very high risk. The literature on consequences of hypoglycaemia specifically relevant to older home-dwelling individuals and the knowledge of diabetes management in order to prevent adverse events, was limited. Future studies addressing hypoglycaemia in older people with diabetes residing at home, are needed, in order to better tailor interventions which can enable them to remain home-dwelling as long as possible.

## Supplementary Information

**Additional file 1: Supplementary file 1.** Medline search strategy (Literature search performed July 2nd 2018).

## Data Availability

The protocol and dataset are available from the corresponding author on reasonable request.

## Abbreviations

CGM: Continuous glucose monitoring
DM: Diabetes mellitus
HbA1c: Glycosylated haemoglobin
T1DM: Type 1 diabetes mellitus
T2DM: Type 2 diabetes mellitus
