# Supplementary material for: Hypoglycaemia in older home-dwelling people with diabetes- a scoping review
Source: BMC Geriatr. 2021 Jan 7;21:20. doi: 10.1186/s12877-020-01961-6 (PMC7792330; doi:10.1186/s12877-020-01961-6)
Supplement: Supplementary file 1 — Additional file 1: Supplementary file 1. Medline search strategy (Literature search performed July 2nd 2018). [file 12877_2020_1961_MOESM1_ESM.docx]

**Supplementary file 1:** Medline search strategy (Literature search performed July 2^nd^ 2018)

1 exp Diabetes Mellitus/ (383844)

2 exp Insulin Resistance/ (72643)

3 (DDM or NIDDM or MODY or T1DM or T2DM or T1D or T2D).tw. (37219)

4 (type 1 diabet* or type i diabet* or type 2 diabet* or type ii diabet*).mp. [mp=title, abstract, original title,

name of substance word, subject heading word, floating sub-heading word, keyword heading word, protocol supplementary concept word, rare disease supplementary concept word, unique identifier, synonyms] (156108)

5 ((non and insulin* and depend*) or (noninsulin* and depend*) or (non and insulin?depend*) or

noninsulin?depend*).tw. (18007)

6 ((insulin* and depend*) or insulin?depend*).tw. (67774)

7 (insulin resistance or glucose intolerance or imparied glucose tolerance).tw. (75139)

8 (insulin* defic* adj relative*).tw. (6)

9 (metabolic syndrome* or plurimetabolic syndrome*).tw. (43146)

10 (nonketotic diabet* or non ketotic diabet* or adult onset diabet* or late onset diabet* or mature diabet* or slow

diabet* or stabil* diabet*).tw. (859)

11 1 or 2 or 3 or 4 or 5 or 6 or 7 or 8 or 9 or 10 (538494)

12 exp Diabetes Insipidus/ (7597)

13 (diabet* and insipidus).tw. (8008)

14 12 or 13 (10227)

15 11 not 14 (537584)

16 Hypoglycemia/ (25405)

17 (hypoclycaemia or hypoglycemia).ti,ab. (26042)

18 16 or 17 (39053)

19 11 and 18 (16609)

20 exp Hypoglycemic Agents/ (228856)

21 ((hypoglycemic or hypoglycaemic or antidiabetes) and (agent* or drug*)).ti,ab. (8030)

22 exp Insulin/ (175853)

23 (carbamoylinsulin or carbamylinsulin or carbonyl bis methionyl insulin or diacetoacetyl insulin ordiacetylinsulin

or diaminosuberoyl insulin or analog insulin or analogue insulin or insulins or methylthiocarbamoylinsulin or methylthiocarbamylinsulin or mononitroinsulin or polyalanylinsulin series or suberoyl insulin or succinyl insulin or triacetylinsulin or tricarbamylinsulin or insulin*).tw. (331671)

24 exp Sulfonylurea Compounds/ (18416)

25 Sulfonylurea Receptors/ (1202)

26 (sulfonurea derivative or sulfonylurea compounds or sulfonylurea series or sulfonylureas, first generation or

sulfonylureas, second generation or sulphonylurea derivative or sulfonylurea receptor*).tw. (1015)

27 20 or 21 or 22 or 23 or 24 or 25 or 26 (410498)

28 15 and 18 and 27 (12956)

29 exp Nursing Homes/ (36537)

30 exp Long-Term Care/ (24417)

31 (nursing homes or long-term care).ti,ab. (30283)

32 29 or 30 or 31 (67788)

33 15 and 18 and 27 and 32 (54)

34 exp Home Care Services/ (44434)

35 Community Health Services/ (29890)

36 ((home or community) adj3 (nursing or care or health service*)).ti,ab. (66745)

37 34 or 35 or 36 (121189)

38 15 and 18 and 27 and 37 (59)

39 33 or 38 (91)

40 aged/ or "aged, 80 and over"/ (2821244)

41 39 and 40 (53)

42 limit 39 to "all aged (65 and over)" (53)

43 41 or 42 (53)
